# Altered functional connectivity during performance feedback processing in multiple sclerosis

**DOI:** 10.1016/j.nicl.2022.103287

**Published:** 2022-12-07

**Authors:** Christopher J. Cagna, Ahmet O. Ceceli, Joshua Sandry, Jamil P. Bhanji, Elizabeth Tricomi, Ekaterina Dobryakova

**Affiliations:** a Department of Psychology, Rutgers University – Newark, 101 Warren Street, Newark, NJ 07102, United States; b Department of Psychiatry, Icahn School of Medicine at Mount Sinai, 1 Gustave L. Levy Place, New York, NY 10029, United States; c Department of Psychology, Montclair State University, 1 Normal Avenue, Montclair, NJ 07043, United States; d Center for Traumatic Brain Injury Research, Kessler Foundation, 120 Eagle Rock Avenue, East Hanover, NJ 07936, United States

**Keywords:** Multiple sclerosis, fMRI, Feedback, Cognitive fatigue, Reward, Striatum

## Abstract

Effective learning from performance feedback is vital for adaptive behavior regulation necessary for successful cognitive performance. Yet, how this learning operates in clinical groups that experience cognitive dysfunction is not well understood. Multiple sclerosis (MS) is an autoimmune, degenerative disease of the central nervous system characterized by physical and cognitive dysfunction. A highly prevalent impairment in MS is cognitive fatigue (CF). CF is associated with altered functioning within cortico-striatal regions that also facilitate feedback-based learning in neurotypical (NT) individuals. Despite this cortico-striatal overlap, research about feedback-based learning in MS, its associated neural underpinnings, and its sensitivity to CF, are all lacking. The present study investigated feedback-based learning ability in MS, as well as associated cortico-striatal function and connectivity. MS and NT participants completed a functional magnetic resonance imaging (fMRI) paired-word association task during which they received trial-by-trial monetary, non-monetary, and uninformative performance feedback. Despite reporting greater CF throughout the task, MS participants displayed comparable task performance to NTs, suggesting preserved feedback-based learning ability in the MS group. Both groups recruited the ventral striatum (VS), caudate nucleus, and ventromedial prefrontal cortex in response to the receipt of performance feedback, suggesting that people with MS also recruit cortico-striatal regions during feedback-based learning. However, compared to NT participants, MS participants also displayed stronger functional connectivity between the VS and task-relevant regions, including the left angular gyrus and right superior temporal gyrus, in response to feedback receipt. Results indicate that CF may not interfere with feedback-based learning in MS. Nonetheless, people with MS may recruit alternative connections with the striatum to assist with this form of learning. These findings have implications for cognitive rehabilitation treatments that incorporate performance feedback to remediate cognitive dysfunction in clinical populations.

## Highlights

• Cognitive fatigue does not impact learning from feedback in multiple sclerosis (MS).
• Cortico-striatal regions are activated during feedback processing in MS.
• Enhanced connectivity between striatal and task-relevant regions also occurs in MS.
• MS may engage alternative striatal connections to aid feedback-based learning.

## 1 Introduction

Multiple sclerosis (MS) is an autoimmune, neurodegenerative disease that targets both white and gray matter in the central nervous system (Pitteri et al., 2021, Tsouki and Williams, 2021). The resulting inflammation disrupts neural transmission, which produces a variety of physical and cognitive symptoms. Relapsing-remitting MS (RRMS), the most common phenotype, is characterized by periods of symptom activity (i.e., relapses) followed by periods of remission, during which symptoms diminish or completely disappear (Lublin et al., 2014).

A common debilitating symptom in MS is fatigue, with cognitive fatigue (CF) showing prevalence estimates as high as 90 % (Dobryakova et al., 2017, Manjalay et al., 2019). CF is characterized as difficulty, or the inability, to initiate or sustain mental effort (Chaudhuri & Behan, 2000). It is frequently reported as overwhelming exhaustion that interferes with cognitive functioning (Chaudhuri and Behan, 2000, Chiaravalloti and DeLuca, 2008, Dobryakova et al., 2013, Finke et al., 2015). Two principal forms of CF are state fatigue and trait fatigue. The former refers to dynamic fluctuations in CF in response to an effortful stimulus (e.g., a cognitive task), while the latter refers to “baseline” CF that remains relatively stable over time (Enoka et al., 2021, Genova et al., 2013, Spiteri et al., 2019). CF has been shown to influence various cognitive capacities in MS, including attention, information processing, task-switching, and working memory (Sandry et al., 2014). These findings are mixed, however. Some studies have linked elevated CF to poor task performance, suggesting that CF may interfere with cognitive performance in MS (e.g., Claros-Salinas et al., 2010, Engström et al., 2013, Pokryszko-Dragan et al., 2016). Conversely, other studies have demonstrated no such impact on performance, suggesting that people with MS may engage compensatory mechanisms to buffer, or circumvent, CF to sustain performance (e.g., DeLuca et al., 2008, Genova et al., 2013, Morrow et al., 2009).

Within the brain, CF is linked to altered functioning of cortico-striatal regions – including the ventral striatum (VS), caudate nucleus of the dorsomedial striatum, and prefrontal regions (Dobryakova et al., 2017, Jaeger et al., 2019, Spiteri et al., 2019). Altered activation patterns within striatal regions (Berard et al., 2019, DeLuca et al., 2008, Genova et al., 2013) and altered connectivity between striatal and cortical regions, have been linked to CF in MS. For example, Finke et al. (2015) reported a significant association between greater self-reported fatigue in MS participants and diminished functional connectivity between the caudate and medial prefrontal and orbitofrontal cortices. Similar relationships have been reported in resting-state studies (Finke et al., 2015, Jaeger et al., 2019, Tijhuis et al., 2021), and also during experimental task paradigms (e.g., working memory tasks, Chen et al., 2020a), further underscoring the central role of cortico-striatal functioning in CF in MS.

These same cortico-striatal regions and connections also overlap with circuitry that regulates reward processing, reinforcement learning, and goal-directed behavior in NT individuals (Haber and Knutson, 2010, Haber, 2016, Liljeholm and O’Doherty, 2012). The VS, caudate, and their prefrontal connections (e.g., ventromedial prefrontal cortex) are sensitive to monetary (extrinsic) and non-monetary (intrinsic) rewards and punishments (Aron et al., 2004, Delgado et al., 2000, Delgado et al., 2003, Dobryakova et al., 2017b), including performance-related feedback (Dobryakova and Tricomi, 2013, Shohamy et al., 2004, Shohamy, 2011, Tricomi et al., 2006, Tricomi and Fiez, 2008, Tricomi and Fiez, 2012). This feedback shapes task learning via instrumental outcomes that signal correct or incorrect responses (i.e., positive or negative feedback, respectively), which enables effective regulation of goal-directed behavior to ensure successful performance.

While cognitive impairment across various domains has been reported in MS (Bora et al., 2016, Chiaravalloti and DeLuca, 2008, Genova et al., 2015, Korakas and Tsolaki, 2016, Patti, 2009), there is currently no research on whether learning from feedback is impaired, nor on the neural mechanisms that govern this capacity in MS. This is an important gap to address, given the susceptibility of people with MS to CF, which may impact their ability to learn, and the tendency for rehabilitation settings to use behavioral interventions that employ performance feedback to remediate cognitive impairment caused by disease or injury (Dardiotis et al., 2018, Hart et al., 2019, Whyte et al., 2019). Given the striatum’s importance in feedback-based learning, we postulate that the VS, caudate, and their prefrontal connections may be candidate regions of this ability in MS.

The present study had two aims – 1) to examine whether state CF disrupts feedback-based learning in people with MS with high trait CF and 2) to investigate whether differences in cortico-striatal activity and connectivity exist between MS and NT individuals during feedback processing. Groups of RRMS and NT participants completed an fMRI paired-word association task (Tricomi et al., 2006, Tricomi and Fiez, 2008), during which trial-by-trial performance feedback was provided during a learning phase, and associative memory performance was assessed during a test phase. Participants also provided state fatigue ratings throughout the learning phase. To account for prior mixed findings regarding CF’s impact on performance in MS, we formulated two alternative behavioral hypotheses. We hypothesized that MS participants would either 1) display worse learning between the learning phase and the test phase (i.e., make more associative memory errors during the test phase) than the NT group, as a result of CF directly interfering with feedback processing during the learning phase; or 2) that MS participants would display learning comparable to NTs, as a result of compensatory mechanisms occurring during feedback processing to counteract CF and successfully facilitate learning. Irrespective of the learning effect observed, we also predicted alterations in VS and caudate activity and in cortico-striatal connectivity in the MS group, given the robust association between these regions and CF in MS (ARM et al., 2019).

## 2 Materials and methods

### 2.1 Participants

All participant recruitment, data collection, and fMRI data acquisition took place at Kessler Foundation (East Hanover, New Jersey, USA; West Orange, New Jersey, USA). Participants were screened prior to study enrollment and were excluded if they met any of the following criteria: left-handedness; diagnosis of a neurological disease other than MS (e.g., epilepsy); significant history of alcohol, drug abuse, or psychiatric issues; current diagnosis of major depressive disorder, schizophrenia, or bipolar disorder; and use of steroids, benzodiazepines, or neuroleptics within the past four weeks. MS participants were excluded if they experienced an exacerbation of symptoms (i.e., a relapse) within the past four weeks. Additionally, MS participants were eligible to participate if they reported a raw score of 36 or higher on the Fatigue Severity Scale (FSS; Krupp et al., 1989), a well-established measure of the impact of fatigue on everyday functioning. Such scores indicate significant fatigue, which allowed us to capture trait fatigue effects. Due to challenges with MS recruitment, we were unable to achieve this criterion with every member of the MS sample. Eight MS participants reported scores below our criterion (Table 1). We standardized these participants’ FSS scores to ensure they were not outliers (defined as *z* < -2.58) compared to the rest of the sample enrolled with the criterion. We confirmed they were not and included them in the MS sample.

**Table 1:** Study Participant Demographic Characteristics.

Demographics	MS (n = 29)	NT (n = 28)	Sig.
Age (Years)	44.90 (7.72)	43.25 (6.77)	*t*(55) = -0.86, *p* =.40

Sex (Female/Male)	26/3 (90 % female)	20/8 (71 % female)	*X*^2^(1) = 3.04, *p* =.08

Education Level (Years)	15.48 (2.44)	15.64 (2.20)	t(55) = 0.26, *p* =.80

Time Since Diagnosis (Years)	14.45 (8.18) Mdn = 14.00 IQR = 13.50	N/A	N/A

EDSS	Mdn = 4.00 IQR = 2.50	N/A	N/A

**FSS**	**45.59 (14.44)**	**25.54 (12.26)**	***t(*55) = -5.64, *p* <.001**

Participants Above Trait CF Threshold (FSS ≥ 36)	21/29 (72 %)	6/28 (21 %)	N/A

Total Lesion Volume	7.12 (7.61) Mdn = 4.34 IQR = 7.30	N/A	N/A

Number of Lesions	20.24 (13.60) Mdn = 20 IQR = 16	N/A	N/A

MS = Multiple Sclerosis; NT = Neurotypical; Sig. = Significance; EDSS = Expanded Disability Status Scale; FSS = Fatigue Severity Scale; N/A = Not Applicable. Unless otherwise noted, all values are presented as M(SD) = mean (standard deviation). Mdn = median; IQR = interquartile range.

A sample of 60 individuals – 30 RRMS and 30 NT (age range: 18–55 years) – participated in the study. We limited our MS sample to RRMS, as this disease course is the most common (Dobson and Giovannoni, 2019, Lublin et al., 2014), and because there are differences in cognitive dysfunction and CF between RRMS and other phenotypes (Johnen et al., 2017, Marchesi et al., 2020). Demographic data are provided in Table 1. The final sample consisted of 57 participants (29 MS and 28 NT) after data exclusion (see Supplementary Material for details). RRMS diagnosis was verified through medical records, specifically through McDonald criteria classification. The protocol was approved by the Institutional Review Board of Kessler Foundation. All participants provided informed consent before beginning experimental procedures, and all were compensated for their time.

### 2.2 Experimental design

#### 2.2.1 Experimental paradigm

**Fig. 1:**
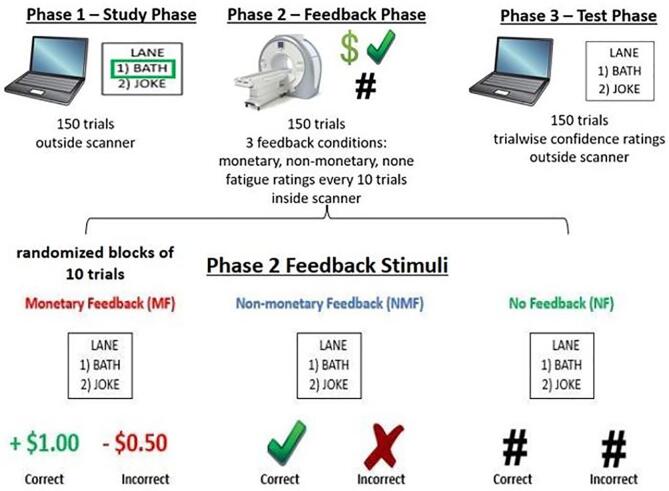
Paired-word association task procedure. Participants first learned associations between arbitrary target words and response options by memorizing the highlighted correct response (Phase 1). They then repeated the same trials in the scanner (Phase 2), but were asked to indicate the correct response (which was no longer highlighted) for each target word. After making their response, they were presented with a feedback stimulus that signaled response accuracy and varied by feedback condition (monetary feedback, non-monetary feedback, or no feedback). During monetary feedback trials, “+$1.00” written in green font was presented after correct responses, and “−$0.50” written in red font was presented after incorrect responses. During non-monetary feedback trials, a green checkmark and red “X” symbol were presented after correct and incorrect responses, respectively. During no feedback trials, a black “#” symbol was presented after every response, regardless of accuracy, thus providing no information about performance. Participants also rated their current cognitive fatigue levels every ten trials using a visual analog scale anchored between 0 (not at all mentally fatigued) and 10 (extremely mentally fatigued). The final phase (Phase 3) served as a test of associative memory, during which feedback was no longer provided. (For interpretation of the references to colour in this figure legend, the reader is referred to the web version of this article.)

The experiment consisted of a modified fMRI version of a feedback-based, paired-associate word learning task (Fig. 1) that has been used in our other work (DiMenichi et al., 2019, Dobryakova and Tricomi, 2013, Tricomi and Fiez, 2008). The task and stimuli were administered with E-Prime (Version 2.0; Psychology Software Tools, Pittsburgh, PA) and consisted of three conditions – *monetary feedback, non-monetary feedback,* and *no feedback*.

Phase 1 (study phase) was completed on a computer outside the scanner and consisted of 150 trials. During each trial, participants viewed a target word and two response options below it. All words were matched for word length, which ranged between four and eight letters, and were also semantically dissimilar from each other. One of the response options was surrounded by a green box, which indicated the correct response for the target word. Participants were instructed to remember the correct word pair. Each trial during Phase 1 lasted four seconds.

fMRI data were acquired during Phase 2 of the task. 150 trials from the study phase were presented again in a randomized order (four seconds per trial) – this time, without a box appearing around the correct response option. Participants were instructed to select the correct paired associate that they learned from the previous phase using one of two buttons. Participants were presented with a feedback stimulus for one second (Fig. 1). There were three feedback conditions: monetary, non-monetary, and no feedback. We included separate monetary and non-monetary conditions to examine possible performance differences in response to extrinsic and intrinsic reward feedback, respectively. During *monetary feedback* trials, a “+ $1.00” stimulus indicating positive feedback appeared after correct responses, and a “- $0.50” stimulus indicating negative feedback appeared after incorrect responses. Participants did not actually win or lose money throughout the task, which was revealed during debriefing. During *non-monetary feedback* trials, a green checkmark was presented after correct responses and a red “X” symbol after incorrect responses. During *no feedback* trials, a black pound symbol (#) was presented after both correct and incorrect responses. Thus, feedback provided information about performance during monetary and non-monetary feedback trials, but not during no feedback trials. After feedback presentation, a jittered fixation appeared 1 to 5 s before the next trial began. All three feedback conditions were presented in randomized blocks of ten trials each. Each participant underwent three functional runs during Phase 2. There were 60 trials each in the monetary and non-monetary feedback conditions, whereas only 30 trials were presented during the no feedback condition to minimize the induction of state fatigue during a condition in which no learning was occurring. To evaluate fatigue severity during task performance, every ten trials, participants rated their current level of cognitive fatigue on the Visual Analogue Scale (VAS-F; Lee et al., 1991) that ranged from 0 (not at all mentally fatigued) to 10 (extremely mentally fatigued).

The final phase (test phase) tested associative memory, and was completed outside the scanner. Participants viewed the same 150 trials in a randomized order and made their selections via keypress. No feedback appeared after their responses. None of the participants verbally reported difficulty performing the task, nor did they display any overt behavioral tendencies indicating a lack of understanding during the task.

#### 2.2.2 Neuropsychological testing

Upon completion of the experimental task, all participants completed a battery of neuropsychological tests (see Supplementary Material for specific tests) to assess group differences in cognitive ability across several domains that could have influenced feedback-based learning during the task. Results are presented in Table 2. Notably, there were no group differences in verbal intelligence, a cognitive domain pertinent for the verbal nature of the experimental task. Additionally, no participants scored lower than 2.58 standard deviations below their group mean verbal intelligence performance, providing further confidence that all participants possessed sufficient cognitive capacity for understanding the task.

**Table 2:** Neuropsychological Test Performance.

Test (Cognitive Domain)	MS n = 29	NT n = 25	Sig.
BVMT-R Immediate Recall (Short-term Visuospatial Learning/Memory)	45.52 (16.55)	49.64 (16.50)	*t*(52) = -0.91, *p* =.37

BVMT-R Delayed Recall (Long-term Visuospatial Learning/Memory)	45.59 (16.62)	50.84 (14.36)	*t*(52) = -1.23, *p* =.22

WASI-II Vocabulary (Verbal Intelligence)	49.31 (12.65)	49.80 (11.24)	*t*(52) = -0.15, *p* =.88

WASI-II Matrix Reasoning (Visuospatial Reasoning)	50.24 (9.73)	49.04 (10.49)	*t*(52) = 0.44, *p* =.66

SDMT (Information Processing)	46.00 (14.62)	51.28 (8.22)	*t*(45.20) = -1.66, *p* =.10

*N*-Back – Total True Positives (Working Memory)	23.45 (2.71)	23.40 (2.78)	*t*(52) = 0.06, *p* =.95

MS = Multiple Sclerosis; NT = Neurotypical; Sig. = Significance; BVMT-R = Brief Visuospatial Memory Test - Revised; WASI-II = Wechsler Abbreviated Scale of Intelligence – Second Edition; SDMT = Symbol Digit Modalities Test. All values are presented as M (SD) = mean (standard deviation). Unless otherwise stated in the variable name, values refer to *t* scores. For independent-samples t tests that violated the homogeneity of variances assumption (assessed by the Levene’s test), degrees of freedom were appropriately corrected before reporting the result.

#### 2.2.3 Post-task questionnaire battery

Participants also completed a series of questionnaires that included measurements of trait fatigue (FSS) and physical disability [Expanded Disability Status Scale (EDSS; Kurtzke, 1983)]. See Supplementary Material for other questionnaires in the battery.

### 2.3 fMRI data acquisition

A 3T Siemens (Erlangen, Germany) MAGNETOM Skyra scanner was used for neuroimaging data acquisition. T1-weighted anatomical images (256 × 256 mm matrix; 1 mm isotropic voxels) were acquired using a Magnetization Prepared Rapid Gradient Echo (MPRAGE) sequence (TR: 2100 ms; TE: 3.43 ms). Thirty-four echo-planar, functional slices were obtained in an interleaved order with the following parameters: 3 mm isotropic voxels, interslice gap: 0.3 mm, TR: 2000 ms, TE: 30 ms, field of view: 204 mm × 204 mm, flip angle: 90°. Functional images were acquired at a transversal orientation to the anterior commissure – posterior commissure (AC-PC) axis. Approximately 255 volumes were acquired during each functional run. VAS-F state fatigue ratings made throughout the task were self-paced, which contributed to slightly different numbers of volumes across runs.

### 2.4 Data analysis

#### 2.4.1 Behavioral data analysis

SPSS statistical software (v. 28.0; IBM Corp., Armonk, NY) and RStudio (v. 4.1.0; R Foundation for Statistical Computing; Vienna, Austria) were used for behavioral data analysis. Independent-samples t tests were used to check for pre-existing group differences in age and education level, while a chi-square test was used to check the same for sex. To confirm that MS participants experienced significantly greater trait fatigue than NT participants, we used an independent-samples *t* test to assess group differences in FSS raw scores.

To examine group differences in learning across the three feedback conditions, while also controlling for any influences of state fatigue, we employed linear mixed effects modeling using the *lme4* package in RStudio. Learning was quantified as the difference in the percentage of correct responses made between Phases 2 and 3 (i.e., Phase 3 performance minus Phase 2 performance, or “delta performance”). Delta performance was entered as the outcome variable. Using no feedback as a reference condition, we dummy-coded two feedback condition fixed effects that estimated differences between the other feedback conditions (monetary feedback, non-monetary feedback) and no feedback. We also included group (MS, NT) as well as group × feedback condition interaction terms as fixed effects. State fatigue ratings (i.e., mean VAS-F scores) for each of the three feedback conditions were included as covariates. We also included a participant random effect. Using the *emmeans* package, post-hoc pairwise comparisons applying the Bonferroni adjustment identified during which feedback conditions learning differed. See Table S2 in the Supplementary Material for model details.

We also tested for differences in subsequent memory performance during Phase 3 as a function of Phase 2 feedback valence (i.e., positive feedback events on correct trials and negative feedback events on incorrect trials), and whether these differences interacted with group. We employed an additional linear mixed effects model with the percentage of Phase 3 correct responses as the outcome variable. Phase 2 feedback valence outcome was dummy-coded (positive as 1, negative as 0) and entered as a fixed effect, as was group and a group × Phase 2 feedback valence interaction term. We also entered state fatigue ratings for the monetary and non-monetary feedback conditions as covariate fixed effects to adjust for state fatigue. Post-hoc pairwise comparisons applying the Bonferroni adjustment were used to identify significant mean differences. See Table S3 in the Supplementary Material for model details.

A Spearman correlation analysis of EDSS scores and delta performance was used to evaluate whether disease-related neurological and functional impairment were associated with MS participants’ learning.

Given MS participants’ susceptibility to fatigue, we also analyzed changes in their state fatigue throughout the task and whether they differed from NT participants. We entered state fatigue rating as the outcome variable. Group and feedback condition were entered as fixed effects. Furthermore, since the order of feedback condition blocks was randomized, we estimated changes in state fatigue across the duration of Phase 2 (i.e., 15 blocks) as a fixed effect. This allowed for the control of cumulative time-on-task on state fatigue ratings. We also included a participant random effect. We modeled both random intercepts and random slopes to account for differences in trait CF at the start of the task, as well as differences in the rate at which state CF occurred throughout the task. See Table S1 in the Supplementary Material for model details.

We also measured possible associations between trait and state CF by conducting Pearson correlation analyses between FSS scores and mean VAS-F ratings within each group for each feedback condition.

Independent-samples t tests using neuropsychological test variables were conducted to assess differences in cognitive ability between MS and NT participants.

#### 2.4.2 fMRI data analysis

A standard pre-processing pipeline using FSL (FMRIB’s Software Library; Version 5.0; Analysis Group, FMRIB, Oxford, United Kingdom) was employed for fMRI pre-processing and analysis. See Supplementary Material for specific parameters.

The time series for outcome valence (i.e., positive feedback, negative feedback) of each condition (i.e., monetary, non-monetary, no feedback) was convolved with a double-gamma canonical hemodynamic response (HRF) function to generate regressors of interest. Corresponding trial onsets for each regressor were concatenated into a single regressor, which was included as a covariate. We modeled trial onsets as a single regressor to minimize collinearity between trial onset and feedback onset events within the model. Additional regressors of no interest included missed trial events, excessive motion outliers, and temporal derivatives of all regressors.

Linear contrasts examined differences in activation between positive and negative feedback events, as well as between each of these events and no feedback. To increase statistical power for these contrasts, we created two additional regressors that estimated activity in response to positive feedback (All Positive Feedback) and negative feedback (All Negative Feedback) by collapsing across monetary and non-monetary feedback conditions. Thus, our main contrasts included All Positive Feedback vs. All Negative Feedback, All Positive Feedback vs. No Feedback, and All Negative Feedback vs. No Feedback. We also included contrasts for comparison to baseline activity. Run-level contrasts of parameter estimates were aggregated to generate subject-level parameter estimates for each of our contrasts, which were used for region-of-interest (ROI) analyses, group-level whole-brain analyses, and generalized psychophysiological interaction (gPPI) analyses.

##### 2.4.2.1 A priori region-of-interest (ROI) analysis

Since prior work has implicated the striatum in performance feedback processing using a similar paradigm (Tricomi and Fiez, 2012, Tricomi and Fiez, 2008), we had a strong *a priori* hypothesis for its recruitment in the current study. We tested this hypothesis within two striatal subregions – the caudate nucleus and the VS. To generate our caudate seed region, two spherical masks using a 5 mm radius (one each in the left caudate and right caudate) were created using MNI coordinates (±13, 10, 11) from an *a priori* caudate seed reported in other studies using similar versions of this task (Lempert and Tricomi, 2015, Tricomi and Fiez, 2012). For the VS seed region, we drew two 5 mm spheres around MNI coordinates (left VS: −10, 11, −8; right VS: 11, 11, −9) from an *a priori* VS seed used in one of those studies (Lempert & Tricomi, 2015). Percent BOLD signal change values were extracted from these seeds for each participant for the following events and contrasts: All Positive Feedback, All Negative Feedback, No Feedback, and All Positive Feedback vs. All Negative Feedback.

Since our central neural hypotheses involved the striatum, we excluded outlier signal to ensure that such extreme values did not lead to potentially false conclusions about striatal activity in response to performance feedback. Percent BOLD signal change values were standardized to *z* scores. Outliers were defined as values less than, or greater than, 2.58 standard deviations from the mean (*z* < −2.58 or *z* > 2.58). Values achieving this criterion comprise the extreme 0.5 % of a normal distribution. Thus, such values were excluded from relevant analyses, which is reflected in the degrees of freedom reported in the results.

For each feedback outcome, single-sample *t* tests assessed whether each striatal region’s activity differed from each group’s respective baseline activation. Independent-samples *t* tests assessed group differences in such activity. To check for group differences in VS and caudate activity, while controlling for activity attributed to CF, we conducted one-way ANCOVAs for each feedback outcome with percent BOLD signal change as the outcome variable, neurological status as the factor, and state fatigue as a covariate.

##### 2.4.2.2 Whole-brain general linear model (GLM) analysis

To identify group activation patterns, subject-level parameter estimates were entered into a mixed-effects model. To account for brain activation uniquely attributed to state fatigue during the task, we also included a demeaned (i.e., mean-centered across groups) state fatigue covariate in the model. A cluster-defining threshold of *z* = 3.1 (α = 0.001) and a cluster-extent threshold of α = 0.017 (0.05/3) were used. This cluster-correction threshold accounted for the three distinct feedback outcomes (All Positive Feedback, All Negative Feedback, No Feedback) for which separate group models were constructed. Group-level linear contrasts identified regions associated with feedback processing during each feedback outcome. We also estimated group differences in fatigue-related BOLD signal during feedback.

##### 2.4.2.3 Generalized psychophysiological interaction (gPPI) analysis

gPPI analysis (McLaren et al., 2012) was conducted to identify regions across the whole brain whose signal temporally correlated with that of our caudate and VS seeds, as a function of feedback valence. Two similar models – one for the caudate seed and one for the VS seed – were constructed for the following feedback outcomes: All Positive Feedback, All Negative Feedback, and All Positive Feedback vs. All Negative Feedback.

To generate the physiological regressor for each model, we transformed our caudate and VS seeds from MNI space to each participant’s native space and then extracted the time series from the resulting native space ROIs. We entered the time series for All Positive Feedback and All Negative Feedback events as our psychological regressors, each convolved with a double-gamma HRF. Temporal derivatives of each psychological regressor were also included. PPI regressors were generated by multiplying each of the zero-centered (FSL, 2014) psychological regressor’s time series with that of the unconvolved physiological regressor. We also modeled corresponding trial onsets of All Positive Feedback and All Negative Feedback events as a single concatenated covariate, which was used to create a corresponding trial onset PPI covariate. Onsets of missed trials and time points identified as motion outliers were included as nuisance regressors. Linear contrasts identified brain regions predicted by the interaction between caudate/VS activity and each feedback type (i.e., PPI for All Positive Feedback and PPI for All Negative Feedback). We compared this connectivity to baseline levels and also assessed differences between positive and negative feedback.

Second-level and group-level analyses followed similar procedures as those outlined in our original GLM. Similar to the whole-brain analysis, we also estimated group differences in state fatigue-related connectivity. We used a cluster-defining threshold of *z* = 3.1 (α = 0.001) and a subsequent corrected threshold of α = 0.013 (0.05/4). This cluster-correction threshold accounted for the two distinct feedback outcomes (All Positive Feedback and All Negative Feedback) for which separate group connectivity models were constructed for the VS seed and caudate seed.

#### 2.4.3 Lesion analysis

The Lesion Segmentation Toolbox of Statistical Parametric Mapping (SPM; Wellcome Centre for Human Neuroimaging; UCL Queen Square Institute of Neurology; London, United Kingdom) was used to measure total lesion volume and number of lesions within the MS sample. This toolbox segments T2 hyperintense lesions by combining information from T1 and FLAIR structural images to generate lesion probability maps. All lesion masks were quality assured. Lesion characteristics of the MS sample are provided in Table 1.

## 3 Results

### 3.1 Group characteristics

**Fig. 2:**
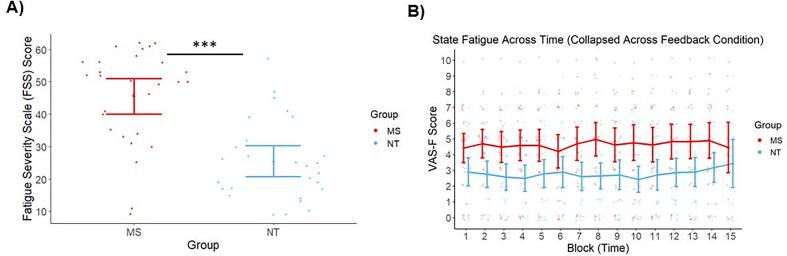
Trait and state fatigue results. A) Raw Fatigue Severity Scale (FSS) scores reported by each group. Maximum possible score is 63. MS participants reported significantly greater trait fatigue than NT participants. B) State fatigue ratings across duration of Phase 2, collapsed across feedback conditions. MS participants reported significantly greater fatigue than NT participants throughout this phase. Fatigue levels remained relatively stable for each group. Individual points denote participant means. Error bars depict 95 % confidence intervals. ***p <.001.

As detailed in Table 1, there were no significant differences in age, sex, and education level between the MS and NT groups (all *p*’s > 0.05). Compared to NT participants, MS participants reported significantly higher scores on the FSS [*t*(55) = 5.64, *p* <.001, *d* = 1.50] (Fig. 2A), indicating greater trait fatigue in this group.

### 3.2 Behavioral results

#### 3.2.1 MS participants reported elevated levels of cognitive fatigue

Likelihood ratio tests revealed a significant contribution of group [*X^2^*(1) = 9.71, *p* =.002] to model fit of the state fatigue data. Parameter estimates revealed that MS participants reported more state fatigue throughout the task, relative to their NT peers (*b* = 1.86, *b_SE_ =* 0.58, *t* = 3.20, *p* =.002). There were no significant contributions of time-on-task [*X^2^*(1) = 1.73, *p* =.19] to model fit, nor of either monetary feedback [*X^2^*(1) = 0.99, *p* =.32] or non-monetary feedback [*X^2^*(1) = 0.57, *p* =.45] compared to no feedback. These results indicate that state fatigue levels remained consistent throughout the task and were not dependent on the type of performance feedback (extrinsic or intrinsic) being provided. The mean state fatigue levels for each block across Phase 2 are displayed in Fig. 2B. Due to there being no condition-specific differences, Fig. 2B is collapsed across feedback conditions.

FSS (i.e., trait fatigue) scores only positively correlated with mean state fatigue scores (averaged across feedback blocks) in the MS group [*r*(26) = 0.50, *p* =.006]. This effect was observed within each feedback condition [monetary feedback: *r*(26) = 0.52, *p* =.004; non-monetary feedback: *r*(26) = 0.48, *p* =.01; no feedback: *r*(26) = 0.48, *p* =.01]. No such association was observed in the NT group [all conditions: *r*(26) = 0.22, *p* =.26; monetary feedback: *r*(26) = 0.26, *p* =.18; non-monetary feedback: *r*(26) = 0.24, *p* =.22; no feedback: *r*(26) = 0.15, *p* =.45].

#### 3.2.2 CF did not disrupt feedback learning and cognitive performance in MS

**Fig. 3:**
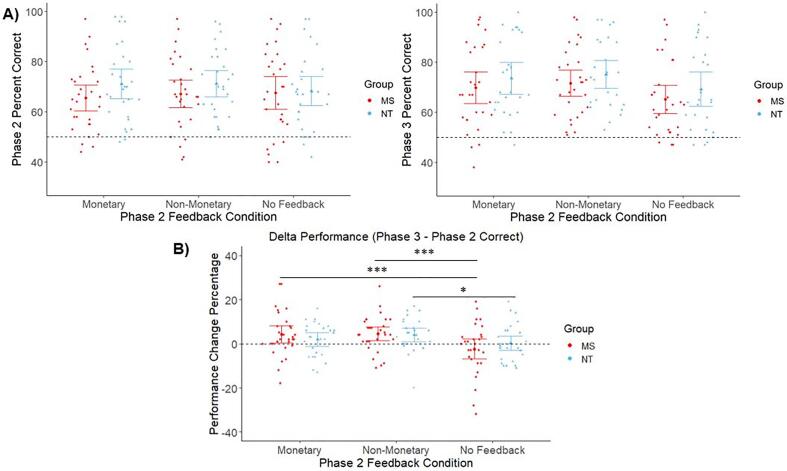
Task performance results. A) Task performance during the feedback phase (Phase 2; left panel) and test phase (Phase 3; right panel) for each group across the three feedback conditions. B) Change in performance between Phase 2 and Phase 3, which served as the measure of learning. Positive values indicate learning between phases. MS participants displayed better learning for word pairs for which they received either monetary or non-monetary performance feedback during Phase 2, compared to word pairs for which they received no feedback. NT participants displayed learning for word pairs for which they previously received non-monetary feedback compared to no feedback. There was no overall difference in learning between the two groups. State fatigue was controlled in this analysis. Note: dotted lines in Panel A denote chance performance (50% response accuracy). Dotted line in Panel B denotes no learning between phases (0% performance change). Individual points denote participant means. Error bars depict 95% confidence intervals. *p <.05; ***p <.001.

Likelihood ratio tests revealed a significant contribution of monetary feedback [*X^2^*(1) = 28.48, *p* <.001] and non-monetary feedback [*X^2^*(1) = 36.61, *p* <.001] to model fit of delta performance data. Parameter estimates indicated successful learning between the feedback phase and test phase (i.e., significantly greater delta performance) for monetary feedback trials (*b* = 0.07, *b_SE_ =* 0.01, *t* = 5.29, *p* <.001) and for non-monetary feedback trials (*b* = 0.07, *b_SE_ =* 0.01, *t* = 5.51, *p* <.001), compared to no feedback trials. We also observed a group × feedback condition interaction [*X^2^*(1) = 7.33, *p* =.007; *b* = -0.05, *b_SE_ =* 0.02, *t* = -2.71, *p* =.007]. Pairwise comparisons revealed that MS participants displayed better learning of word pairs from monetary feedback trials (*p* <.001) and from non-monetary feedback trials (*p* <.001), compared to word pairs from no feedback trials. NT participants displayed better learning of word pairs from non-monetary feedback trials compared to those from no feedback trials (*p* =.03). However, there was no overall difference in learning between the two groups [*X^2^*(3) = 7.55, *p* =.06; *b* = 0.03, *b_SE_ =* 0.02, *t* = 1.38, *p* =.17]. Collectively, these results indicate that both groups successfully learned from performance feedback provided during Phase 2. Although there was no overall difference in learning between groups, MS participants’ learning benefited from both monetary and non-monetary feedback, while NT participants’ learning benefited from non-monetary feedback. Importantly, we observed these effects after adjusting for state fatigue during each feedback condition. State fatigue did not significantly impact learning across groups [monetary feedback state fatigue: *X^2^*(1) = 0.45, *p* =.50; *b* = -0.01, *b_SE_ =* 0.02, *t* = -0.64, *p* =.52; non-monetary feedback state fatigue: *X^2^*(1) = 0.38, *p* =.54; *b* = 0.01, *b_SE_ =* 0.02, *t* = 0.59, *p* =.56; no feedback state fatigue: *X^2^*(1) = 0.09, *p* =.76; *b* = 0.004, *b_SE_ =* 0.01, *t* = 0.29, *p* =.77]. Mean performance during Phase 2 and during Phase 3 for each feedback condition is displayed in Fig. 3A, while the mean change in performance between phases (delta performance) is displayed in Fig. 3B.

**Fig. 4:**
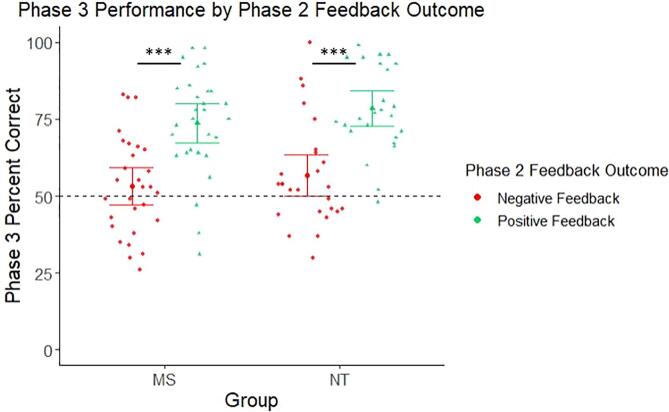
Test phase performance results, as a function of prior feedback valence. Both MS and NT participants correctly recalled more word pairs during Phase 3 for which they previously received positive feedback during Phase 2, compared to word pairs for which they previously received negative feedback. Performance during monetary feedback and non-monetary feedback conditions were collapsed in this analysis. State fatigue was also controlled. Note: dotted line denotes chance performance (50% response accuracy). Individual points denote participant means. Error bars depict 95% confidence intervals. ***p <.001.

With respect to performance at test, likelihood ratio tests revealed a significant contribution of Phase 2 feedback valence to model fit of Phase 3 performance data [*X^2^*(2) = 56.72, *p* <.001]. Parameter estimates indicated that prior positive feedback predicted significantly better performance during Phase 3 (*b* = 0.21, *b_SE_ =* 0.03, *t* = 6.89, *p* <.001), but this effect did not differ by group [*X^2^*(1) = 0.10, *p* =.75; *b* = 0.01, *b_SE_ =* 0.04, *t* = 0.31, *p* =.76] (Fig. 4). These results indicate that both groups’ performance during test particularly benefitted from positive feedback received during Phase 2. Additionally, there were no significant influences of state fatigue on this effect [monetary feedback state fatigue: *X^2^*(1) = 0.16, *p* =.69; *b* = -0.01, *b_SE_ =* 0.03, *t* = -0.39, *p* =.70; non-monetary feedback state fatigue: *X^2^*(1) = 0.06, *p* =.80; *b* = 0.01, *b_SE_ =* 0.03, *t* = 0.24, *p* =.81].

#### 3.2.3 Neuropsychological test performance and EDSS

MS participants did not differ in neuropsychological test performance relative to their NT counterparts, suggesting no pre-existing differences in cognitive capacity that could have influenced feedback-based learning during the task. This is supported in Table 2. Furthermore, as with fatigue, we did not observe any potential negative impact of disability status (measured via EDSS) on learning in any of the feedback conditions [monetary feedback: *r_s_*(27) = 0.02, *p* =.92; non-monetary feedback: *r_s_*(27) = 0.09, *p* =.65; no feedback: *r_s_*(27) = 0.22, *p* =.25]. We observed a similar result when collapsing across delta performance in all conditions: *r_s_*(27) = 0.22, *p* =.26.

### 3.3 fMRI results

#### 3.3.1 MS and NTs recruit similar cortico-striatal regions in response to All Positive Feedback vs All Negative Feedback

Since we did not identify measurable differences in learning between the two feedback conditions (i.e., monetary and non-monetary feedback; Fig. 3), we collapsed across these conditions to form “All Positive Feedback” and “All Negative Feedback” regressors (as described in the Method) used in the whole-brain GLM (See Table 3).

**Table 3:** Whole-brain Analysis Activation Clusters and Local Maxima for Primary Feedback Contrasts.

				MNI Coordinates (mm)		
Cluster Number	Brain Region (Hemisphere)	Cluster Size (Voxels)	Z-statistic	x	y	z
**Whole-Brain Analysis**						
***All Positive Feedback > All Negative Feedback, MS***						
1	Ventral striatum (R)	5957	6.47	14	8	−10
	Ventral striatum (L)		5.91	−8	6	−10
	Putamen (L)		5.79	−16	16	−6
	Caudate (R)		5.61	18	−2	28
	Ventromedial prefrontal cortex (L)		4.98	−2	54	−6
2	Central opercular cortex (L)	744	5.14	−40	−4	18
	Caudate (L)		4.34	−16	4	24
3	Superior lateral occipital cortex (L)	588	4.53	−26	−88	14
	Lingual gyrus (L)		4.29	−14	−76	−4
	Intracalcarine cortex (L)		3.62	−26	−70	12
4	Precuneus (L)	214	4.22	−2	−60	22
5	Cerebellum (R)	182	4.39	40	−66	−38
6	Posterior cingulate cortex (L)	180	4.55	−2	−44	34
	Posterior cingulate cortex (R)		4.03	4	−42	30

***All Positive Feedback > All Negative Feedback, NT***						
1	Ventromedial prefrontal cortex (L)	16,990	6.33	−8	56	−2
	Ventral striatum (R)		6.28	8	10	−8
	Anterior prefrontal cortex (L)		6.10	−6	50	−6
	Putamen (L)		5.82	−14	10	−8
	Precuneus		5.58	0	−58	26

***All Positive Feedback, MS***						
1	Hippocampus (L)	383	5.00	−28	−16	−14
	Posterior temporal fusiform cortex (L)		4.19	−40	−36	−10
2	Occipital pole (L)	248	5.19	−20	−96	6
	Occipital fusiform gyrus (L)		4.32	−20	−88	2
3	Hippocampus (R)	225	4.16	34	−34	−4
4	Amygdala (R)	142	5.98	26	−10	−14
5	Occipital fusiform gyrus (R)	135	5.20	22	−86	−2

***All Positive Feedback, NT***						
1	Hippocampus (L)	3686	5.63	−38	−26	−12
2	Occipital pole (L)	242	5.44	−20	−96	4
3	Amygdala (R)	173	4.42	22	−4	−16
	Hippocampus (R)		4.34	34	−10	−18
4	Amygdala (L)	114	4.44	–22	2	−20
	Anterior parahippocampal gyrus (L)		4.00	−16	2	–22

***All Negative Feedback, MS***						
1	Posterior medial temporal gyrus (R)	140	4.12	50	−28	−6
2	Insula (L)	140	4.24	−36	16	−10
3	Insula (R)	114	4.55	32	20	−16
4	Ventromedial prefrontal cortex (R)	106	4.13	16	62	−12
	Orbitofrontal cortex (R)		3.77	22	64	−2

***All Negative Feedback, NT***						
1	Insula (R)	149	4.82	34	16	−8
2	Occipital pole (L)	123	4.47	−20	−98	2

***No Feedback, MS***						
1	Posterior temporal fusiform cortex (L)	157	4.52	−38	−26	−16
	Hippocampus (L)		4.51	−26	−18	−14
2	Insula (L)	124	4.26	−44	2	−8
	Planum polare (L)		4.08	−44	−12	−4
3	Planum polare (R)	101	4.36	46	2	−6
	Heschl’s gyrus (R)		4.20	48	−8	0

***No Feedback, NT***						
1	Orbitofrontal cortex (R)	174	4.98	22	34	−12
2	Posterior parahippocampal gyrus (L)	106	4.50	−36	−38	−6

Peak intensities within each cluster with z values exceeding the cluster-defining threshold of 3.1 (p <.001, corrected to p <.017) for each event/contrast of events. Each unique cluster identified within the event/contrast is numerically listed in the “Cluster Number” column. When multiple peaks were identified within the same region, the reported coordinates correspond to peak activation with the highest z value. The Harvard-Oxford and Montreal Neurological Institute (MNI) Structural Atlases were used for the identification of anatomical brain region labels. MS = multiple sclerosis, NT = neurotypical, L = left hemisphere, R = right hemisphere.

**Fig. 5:**
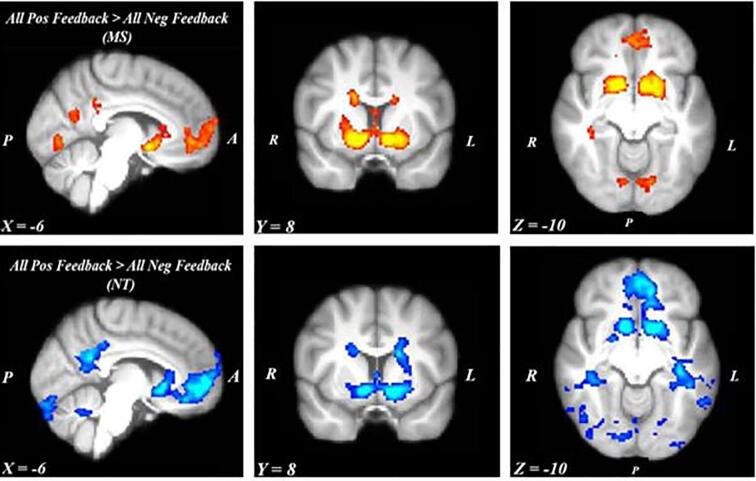
Whole-brain analysis, All Positive Feedback > All Negative Feedback contrast results. Although there were no group differences, MS participants (top row) and NT participants (bottom row) displayed similar functional organization dedicated to the processing of feedback valence. Notably, the largest clusters of activation within each group contained cortico-striatal regions, including the VS and vmPFC. Notes: Images are left–right-reversed. MS activation appears in warmer colors (orange and yellow); NT activation appears in cooler colors (blue). All brain maps used a cluster-defining threshold of α = 0.001 and were cluster-corrected at a threshold of *α* = 0.017. A = anterior; P = posterior; R = right; L = left. Slice numbers are MNI coordinates. (For interpretation of the references to colour in this figure legend, the reader is referred to the web version of this article.)

MS and NT participants recruited largely similar regions in response to All Positive vs. All Negative Feedback. The MS group displayed enhanced activity in striatal regions, including in the bilateral caudate, bilateral VS, and left putamen (Fig. 5). Additional activation was observed in the left central opercular cortex, left lingual gyrus, right cerebellum, left ventromedial prefrontal cortex (vmPFC), and bilateral posterior cingulate. NT participants similarly displayed activity in the right VS and left putamen (Fig. 5). They also displayed activation in the precuneus and left vmPFC. There were no group differences, and there was also no significant brain activity elicited in response to state fatigue.

#### 3.3.2 MS and NTs recruit similar regions in response to All Positive Feedback and All Negative Feedback

In response to All Positive Feedback, both MS and NT participants recruited the amygdala (right for MS and bilateral for NT), bilateral hippocampus, and occipital regions, relative to baseline levels of activity (Table 3). There was no significant difference between groups.

In response to All Negative Feedback, we observed activation in the bilateral insula, right medial temporal gyrus (MTG), right vmPFC, and right orbitofrontal cortex (OFC) for MS participants. NT participants displayed activation in the right insula and in the left occipital pole (Table 3). There was no significant difference between groups.

In both groups, significant clusters of activation in response to state fatigue emerged within the left temporal occipital fusiform cortex and left occipital fusiform gyrus during All Positive Feedback and within the left postcentral gyrus during All Negative Feedback (Table 4). There were no group differences in fatigue-induced regional activation.

**Table 4:** Activation Clusters and Local Maxima for Effects of State Fatigue.

				MNI Coordinates (mm)		
Cluster Number	Brain Region (Hemisphere)	Cluster Size (Voxels)	Z-statistic	x	y	z
**Whole-Brain Analysis**						
***All Positive Feedback – Fatigue (MS and NT)***						
1	Temporal occipital fusiform cortex (L)	142	3.94	−38	−46	−16
	Occipital fusiform gyrus (L)		3.69	−40	−64	−12

***All Negative Feedback – Fatigue (MS and NT)***						
1	Postcentral gyrus (L)	260	4.68	−34	−30	38

Peak intensities within each cluster with z values exceeding the cluster-defining threshold of 3.1 (p <.001, corrected to p <.017) for each event/contrast of events. Each unique cluster identified within the event/contrast is numerically listed in the “Cluster Number” column. When multiple peaks were identified within the same region, the reported coordinates correspond to peak activation with the highest z value. The Harvard-Oxford and Montreal Neurological Institute (MNI) Structural Atlases were used for the identification of anatomical brain region labels. MS = multiple sclerosis, NT = neurotypical, L = left hemisphere, R = right hemisphere.

#### 3.3.3 MS and NTs recruit VS and caudate similarly during feedback

**Fig. 6:**
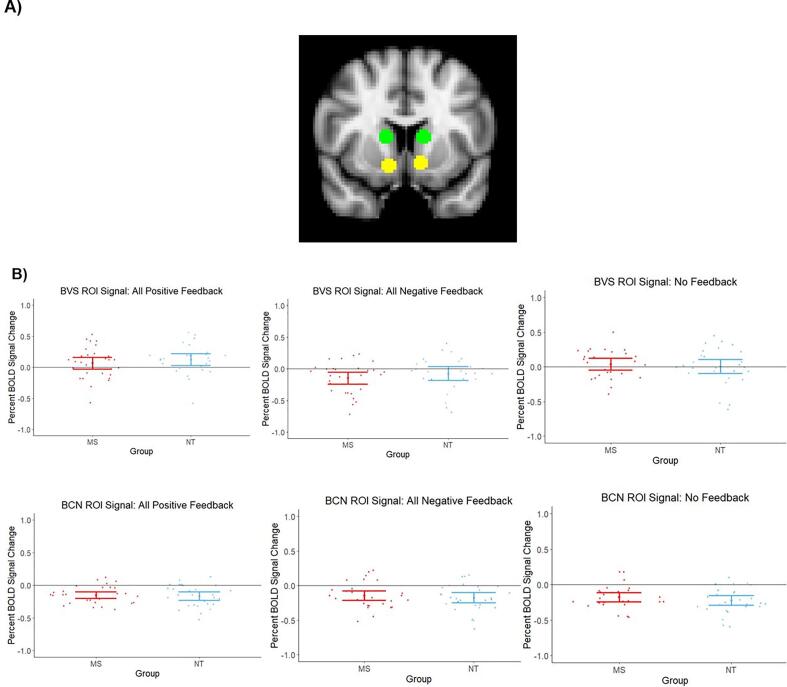
*A priori* region-of-interest analysis results. A) Bilateral caudate (green) and bilateral ventral striatum (yellow) seed regions used for analysis. Caudate seed derived from *a priori* coordinates reported in Lempert and Tricomi (2015). MNI coordinates: ± 13, 10, 11. Ventral striatum seed derived from *a priori* coordinates reported in Lempert & Tricomi (2015). MNI coordinates: −10, 11, −8 (left); 11, 11, −9 (right). B) VS (top row) and caudate (bottom row) signal during primary feedback outcomes. Overall, the VS shows more sensitivity to feedback valence, while the caudate shows more generalized reductions in activity across feedback outcomes. There were no group differences in activity within either region, suggesting similar engagement of the striatum during feedback processing between MS and NT participants. All results were controlled for state fatigue. Individual points denote participant means. Error bars depict 95% confidence intervals. BVS = bilateral ventral striatum; BCN = bilateral caudate nucleus. (For interpretation of the references to colour in this figure legend, the reader is referred to the web version of this article.)

There were no group differences in bilateral VS seed region activity (Fig. 6A) for each feedback outcome. MS participants displayed significantly stronger VS activity during All Positive Feedback, compared to All Negative Feedback: [*t*(27) = 8.67, *p* <.001, *d* = 1.64], reflecting the strong cortico-striatal activation observed for this same contrast in our whole-brain analysis. Relative to baseline, MS participants did not display significant VS activity in response to All Positive Feedback [*t*(28) = 1.46, *p* =.16, *d* = 0.27], but did display significantly decreased activity during All Negative Feedback [*t*(28) = -3.17, *p* =.004, *d* = -0.59] (Fig. 6B).

NT participants, however, displayed significant increases in activity, relative to baseline, during All Positive Feedback [*t*(26) = 2.78, *p* =.01, *d* = 0.54], but no significant reductions in activity in response to All Negative Feedback [*t*(25) = -1.39, *p* =.18, *d* = -0.27]. Similar to the MS group, NT participants displayed greater VS activity during All Positive, relative to All Negative, Feedback: [*t*(27) = 6.30, *p* <.001, *d* = 1.19] – also reflecting the results observed for this group in the whole-brain analysis (Fig. 6B).

Neither group showed significant VS activity during No Feedback outcomes: [MS: *t*(25) = 1.00, *p* =.33, *d* = 0.20; NT: *t*(27) = 0.17, *p* =.87, *d* = 0.03] (Fig. 6B).

We conducted the same analysis for the bilateral caudate seed region (Fig. 6A). Relative to baseline levels, the MS group displayed decreased activity in response to All Positive Feedback [*t*(27) = −5.80, *p* <.001, *d* = −1.10]; All Negative Feedback [*t*(28) = −4.25, *p* <.001, *d* = −0.79]; and No Feedback [*t*(25) = −5.46, *p* <.001, *d* = −1.07]. MS participants also did not display differential caudate activity in response to All Positive Feedback compared to All Negative Feedback outcomes [*t*(27) = 0.94, *p* =.36, *d* = 0.18] (Fig. 6B).

The NT group displayed similar decreases in caudate activity, relative to baseline, in response to All Positive Feedback [*t*(27) = −5.23, *p* <.001, *d* = −0.99]; All Negative Feedback [*t*(27) = −4.83, *p* <.001, *d* = −0.91]; and No Feedback [*t*(27) = −6.57, *p* <.001, *d* = −1.24] (Fig. 6B). Interestingly, NT participants also did not display differential caudate activity during All Positive vs. All Negative Feedback [*t*(27) = 1.49, *p* =.15, *d* = 0.29]. This result was unexpected in the NT group, since the caudate has displayed such sensitivity in previous work (Tricomi & Fiez, 2012). A voxelwise GLM analysis of this same contrast restricted to the same bilateral caudate seed region revealed significant activation within a subset of voxels in the left caudate in the NT group after applying a more liberal cluster-defining threshold (*α* = 0.01). In addition, activation was present in a more ventral portion of the caudate – towards the VS and outside of our caudate seed – in the whole-brain GLM for this contrast at a more stringent threshold (*α* = 0.001). Thus, we replicate caudate sensitivity to positive vs. negative feedback in our NT sample, albeit to a weaker degree than in previous work. There were no significant differences in caudate activity between the two groups for any feedback outcome.

ANCOVA analyses revealed no impact of state fatigue on either VS signal [All Positive Feedback: *F*(1,53) = 1.56, *p* =.22, η^2^ = 0.03; All Negative Feedback: *F*(1,52) = 1.92, *p* =.17, η^2^ = 0.04; No Feedback: *F*(1,52) = 1.36, *p* =.25, η^2^ = 0.03] or caudate signal [All Positive Feedback: *F*(1,52) = 0.89, *p* =.35, η^2^ = 0.02; All Negative Feedback: *F*(1,52) = 3.20, *p* =.08, η^2^ = 0.06; No Feedback: *F*(1,52) = 0.01, *p* =.93, η^2^ < 0.001].

#### 3.3.4 Connectivity in response to All Positive Feedback in MS participants

gPPI results revealed no significant changes in either VS or caudate connectivity during All Positive vs. All Negative Feedback within either group.

**Fig. 7:**
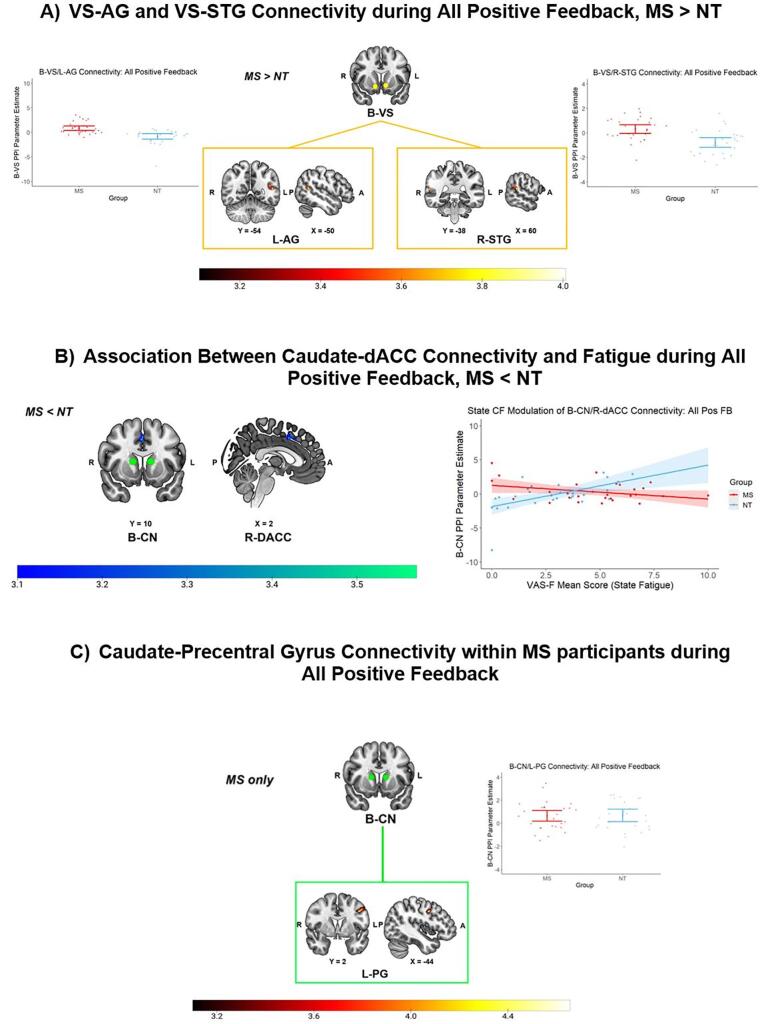
Functional connectivity results. A) gPPI results revealed stronger connectivity between the VS (yellow circles, top panel), left AG (left panel), and right STG (right panel) in the MS group, compared to the NT group, during All Positive Feedback. B) Relative to NT participants, MS participants displayed potential reductions in connectivity between the caudate (green circles) and right dACC, in response to state fatigue experienced during All Positive Feedback, though the cluster size associated with this effect does not survive multiple comparison correction. C) Results also revealed stronger connectivity between the caudate (green circles) and left precentral gyrus within MS participants during All Positive Feedback. A cluster-defining threshold of *z* = 3.1 (α = 0.001) and a cluster-extent correction of *α* = 0.013 were applied during all analyses. Warmer colors correspond to enhanced connectivity, while cooler colors correspond to reduced connectivity. Corresponding plots next to each contrast map correspond to participant mean PPI beta coefficient values, along with group level means and 95% confidence intervals, for that contrast. MS = multiple sclerosis; NT = neurotypical; B-VS = bilateral ventral striatum; L-AG = left angular gyrus; R-STG = right superior temporal gyrus; B-CN = bilateral caudate nucleus; R-DACC = right dorsal anterior cingulate cortex; L-PG = left precentral gyrus; R = right; L = left; A = anterior; P = posterior. Slice numbers refer to MNI coordinates. (For interpretation of the references to colour in this figure legend, the reader is referred to the web version of this article.)

When examining All Positive Feedback separately, we observed a group difference, such that the VS displayed stronger connections with the left angular gyrus (AG) and right superior temporal gyrus (STG) in the MS group (Fig. 7A). Relative to baseline, the NT group showed reduced connectivity between the VS and right AG and right STG. We also observed reductions in VS connectivity in response to All Negative Feedback in the NT group. Reduced coupling was observed between the VS and right frontal operculum, right inferior frontal gyrus, and right insula, in addition to prefrontal regions, including the right dorsomedial prefrontal cortex (dmPFC), bilateral dACC, and left ventral ACC (Table 5). There were no changes in VS connectivity associated with state fatigue.

**Table 5:** gPPI Analysis Activation Clusters and Local Maxima for Primary Feedback Contrasts.

				MNI coordinates (mm)		
Cluster Number	Brain region (Hemisphere)	Cluster size (Voxels)	Z-statistic	x	y	z
**gPPI Analysis: Bilateral Caudate Seed**						
***All Positive Feedback, MS, Enhanced Connectivity***						
1	Precentral gyrus (L)	236	4.72	−44	2	38
	Middle frontal gyrus (L)		3.47	−50	8	42

***All Positive Feedback, NT, Reduced Connectivity***						
1	Anterior prefrontal cortex (R)	679	4.38	22	64	16
	Dorsomedial prefrontal cortex (R)		3.93	10	48	44
	Dorsolateral prefrontal cortex (R)		3.85	16	50	30
2	Middle frontal gyrus (R)	311	4.29	32	30	46
	Superior frontal gyrus (R)		4.11	20	20	58
3	Ventromedial prefrontal cortex (L)	163	4.68	−26	54	8
	Anterior prefrontal cortex (L)		3.90	−36	56	0
4	Insula (R)	109	4.99	34	10	0

gPPI Analysis: Bilateral VS Seed						
***All Positive Feedback, MS > NT Connectivity***						
1	Angular gyrus (L)	180	4.03	−50	−54	14
	Medial occipitotemporal gyrus (L)		3.67	−52	−60	6
2	Superior temporal gyrus (R)	102	4.07	60	−38	14
	Angular gyrus (R)		3.50	60	−50	16
***All Positive Feedback, NT, Reduced Connectivity***						
1	Angular gyrus (R)	220	4.57	60	−50	18
	Superior temporal gyrus (R)		3.94	60	−38	12
***All Negative Feedback, NT, Reduced Connectivity***						
1	Frontal operculum cortex (R)	229	4.02	44	18	2
	Insula (R)		3.98	42	4	−8
	Inferior frontal gyrus, pars triangularis (R)		3.64	40	32	−4
2	Dorsomedial prefrontal cortex (R)	177	4.14	4	36	30
	Dorsal anterior cingulate cortex (L)		3.72	−2	24	34
	Dorsal anterior cingulate cortex (R)		3.55	6	26	26
	Ventral anterior cingulate cortex (L)		3.55	−6	32	18
3	Dorsal anterior cingulate cortex (L)	113	3.86	−4	46	−4
	Dorsal anterior cingulate cortex (R)		3.69	8	42	2

Peak intensities within each cluster with z values exceeding the cluster-defining threshold of 3.1 (p <.001, cluster-corrected to p <.013) for each event/contrast of events. Each unique cluster identified within the event/contrast is numerically listed in the “Cluster Number” column. When multiple peaks were identified within the same region, the reported coordinates correspond to peak activation with the highest z value. The Harvard-Oxford and Montreal Neurological Institute (MNI) Structural Atlases were used for the identification of anatomical brain region labels. MS = multiple sclerosis, NT = neurotypical, L = left hemisphere, R = right hemisphere.

During All Positive Feedback, a group interaction emerged, whereby the MS group displayed a reduction in connectivity between the caudate and right dACC that was associated with greater state fatigue, while the NT group displayed increased caudate-dACC connectivity associated with greater state fatigue (Fig. 7B). This interaction also remained after the exclusion of an outlier within the NT group (*b* = 0.62, *b*_SE_ = 0.17, *t* = 3.59, *p* =.001; inclusion of outlier: *b* = 0.81, *b*_SE_ = 0.20, *t* = 3.99, *p* <.001) (see Figure S1 in the Supplementary Material for a visual comparison of plots). As an additional check, we used the *robustbase* package in RStudio to conduct a subsequent robust regression analysis (which is not as sensitive to the influences of outliers) of this relationship within the peak of the dACC cluster, which revealed a sustained significant group interaction when assuming a normal t distribution (*b* = 0.58, *b*_SE_ = 0.20, *t* = 2.90, *p* =.005; exclusion of outlier: *b* = 0.58, *b*_SE_ = 0.20, *t* = 2.85, *p* =.006). However, the associated cluster size did not survive correction for multiple comparisons for the number of connectivity models (α = 0.013). Thus, we advise caution with interpretation of this effect. Within-group analyses revealed a non-significant trending association between reduced caudate-dACC connectivity and greater state fatigue in MS participants [*r*(27) = −0.34, *p* =.08]. In NT participants, exclusion of the outlier did not influence the significant association between enhanced caudate-dACC connectivity and greater state fatigue [outlier included: *r*(26) = 0.56, *p* =.002; outlier excluded: *r*(25) = 0.54, *p* =.004]. These results suggest that this potential differential impact of state CF on caudate-dACC connectivity across groups was driven by a stronger relationship within the NT group.

With respect to within-group connectivity, the MS group displayed enhanced connections between the caudate and left precentral gyrus and left middle frontal gyrus (Fig. 7C). Diminished connectivity with the right vmPFC was also observed, but did not survive correction for multiple comparisons between models (See Fig. S2 in the Supplementary Material). The NT group showed reduced connectivity between the caudate and prefrontal regions, including the right dmPFC, right dlPFC, and left vmPFC. Reduced connectivity was also observed between the caudate and right medial frontal gyrus, right superior gyrus, and right insula in this group (Table 5). There were no group differences in caudate connectivity associated with All Positive Feedback. We also did not observe any differences during All Negative Feedback outcomes – in response to either feedback or fatigue.

We did not observe any significant associations between any of these connectivity patterns and learning between Phase 2 and Phase 3 (i.e., delta performance). However, within the MS group, a non-significant trending association emerged between strengthened VS-STG connectivity and better learning: *r*(27) = 0.34, *p* =.07.

## 4 Discussion

Despite MS participants reporting greater trait and state fatigue, they performed comparably to their NT peers, displaying similar patterns of better associative memory performance after receiving performance feedback. After accounting for state CF, we found similar patterns of cortico-striatal recruitment – largely within the VS caudate, and vmPFC – in both groups during feedback processing. Functional connectivity analyses, however, revealed group differences in striatal connectivity. MS participants displayed more enhanced connectivity between the VS and left AG and between the VS and right STG, compared to NT participants. Results also indicate potential reductions in connectivity between the caudate and dACC in response to CF during positive feedback outcomes in MS participants. Collectively, these results suggest that CF might influence cortico-striatal connectivity in MS during feedback processing, but not to a degree that interferes with learning from feedback. However, people with MS may instead recruit alternative striatal connections to assist with this form of learning.

### 4.1 Self-reported cognitive fatigue and cognitive performance

Both MS and NT participants correctly recalled more word pairs previously learned with informative feedback, compared to no feedback, suggesting that learning within both groups benefited from performance feedback. Interestingly, this comparable task performance was accompanied by higher self-reported trait fatigue and state fatigue in the MS group. These results support our second alternative behavioral hypothesis – namely, that CF does not interfere with feedback-based learning and subsequent cognitive performance in MS. These findings are consistent with other reports of no negative impact of CF on performance within other domains – such as processing speed (DeLuca et al., 2008, Sandry et al., 2014), task-switching (Genova et al., 2013), attention and vigilance (Hu et al., 2019), and working memory (Morrow et al., 2009, Sandry et al., 2014).

One possible explanation for the MS group’s effective performance is a compensatory mechanism that enabled participants to sustain cognitive performance in spite of their CF. Indeed, it has been suggested that disparities between self-reported CF and objective task performance in MS are the result of compensatory, but not necessarily efficient, functional changes within the brain (Chen et al., 2020b, Chiaravalloti et al., 2015, DeLuca et al., 2008, Manjalay et al., 2019). That is, functional reorganization in response to mobilization of cognitive resources needed for effective task performance may not occur in the most adaptive manner (Chiaravalloti et al., 2015, Schoonheim and Filippi, 2012, Schoonheim, 2017). This suboptimal reorganization may promote satisfactory performance, but at the expense of additional “cerebral resources'' (as stated in Chen et al., 2020b). Consumption of these excessive resources is then manifested as subjective CF, while task performance remains unperturbed (DeLuca et al., 2008).

### 4.2 Functional connectivity group differences in response to positive feedback

Group differences in functional connectivity during performance feedback processing in the current study may provide some support for this explanation. Compared to NT participants, MS participants showed significantly stronger coupling between the VS and left AG and right STG, in response to positive feedback. Stronger VS-STG connectivity also displayed a non-significant trending association with better learning within the MS group. The left AG is associated with semantic retrieval of language (Binder et al., 2009, Davey et al., 2015, Seghier, 2013). The right STG is associated with auditory processing and language comprehension (Bhaya-Grossman & Chang, 2022), and has been implicated in insight-based problem-solving during a word-learning task (Jung-Beeman et al., 2004). Thus, it is possible that strengthened connections with the AG and STG served as compensatory reorganization that enabled MS participants to effectively learn from feedback about word pairs via recruitment of alternate striatal connections with task-relevant regions. This may have aided MS participants’ favorable task performance. This possibility is further supported by MS participants’ significantly better test performance on trials for which they previously received positive feedback (the same trials during which connectivity changes were also observed), compared to trials for which they previously received negative feedback. This finding also aligns with other work reporting beneficial associations between positive feedback (and other rewards) and task performance (Lempert and Tricomi, 2015, Tricomi and Fiez, 2012, Wӓchter et al., 2009). However, future replication with a larger sample is warranted, given the large number of participants that may be required to detect such brain-behavior relationships (Marek et al., 2022).

Our results also indicate potential attenuated coupling between the caudate and dACC associated with higher state fatigue ratings in MS participants, compared to NT participants, in response to positive feedback. The dACC is associated with performance monitoring and cognitive control functions (Brockett et al., 2020, Quilodran et al., 2008, Sheth et al., 2012), which are essential for effectively learning from feedback information and flexibly adapting behavior. However, there were no significant relationships between caudate-dACC connectivity and learning in the current study. An alternative explanation is that the reduced caudate-dACC connectivity in the MS group was attributed to state CF dampening *subjective value* of feedback. This may explain why connectivity findings were only observed during positive feedback. The dACC plays a role in cost-benefit computations that yield subjective value (Croxson et al., 2009). Given that the caudate responds to reward value (Delgado et al., 2000, Delgado et al., 2003, Tricomi et al., 2006, Tricomi and Fiez, 2008) and regulates motivated action towards perceived reward (Grahn et al., 2008), it is possible that diminished coupling with the dACC contributed to a cost-reward imbalance that disrupted subjective valuation of positive feedback in the MS group, but otherwise did not impact their learning from feedback. However, whole brain analysis results suggest that this was not a complete attenuation, as we did observe greater VS and caudate activation to positive, relative to negative, feedback in the MS group. Furthermore, we advise caution with this potential explanation, as the dACC cluster associated with this group interaction effect did not survive the more stringent correction threshold we employed (α = 0.013) to control for multiple comparisons.

Within the NT group, there was a significant relationship between greater state CF and enhanced connectivity between these regions that was robust to the exclusion of an outlier. These findings align with reports of a similar relationship between enhanced cortico-striatal connectivity and elevated CF during a demanding working memory task in NT individuals (Chen et al., 2020a). Within the MS group, a non-significant trending relationship between greater CF and diminished caudate-dACC connectivity was observed. It is possible that inter-subject variability from influences of disease-specific mechanisms contributing to CF in the MS group (e.g., variability in inflammatory response patterns) may have also obscured the relationship between cortico-striatal hypoconnectivity and state CF in the current study.

Thus, the current study provides evidence for altered VS functional connectivity during performance feedback processing in people with MS that may assist their learning from that feedback. Additional work, however, is needed to further probe the nature of the relationship between caudate functional connectivity and CF that occurs during feedback processing in MS.

### 4.3 Common cortico-striatal region recruitment between groups in response to feedback

Our results also highlight a set of common cortico-striatal regions recruited by both MS and NT participants during the processing of feedback valence. After adjusting for state CF, whole-brain GLM results suggest that individuals with MS show similar modulation of cortico-striatal circuitry – comprising the VS caudate, and vmPFC – by feedback valence. In line with reports of these regions’ sensitivity to feedback valence (Delgado et al., 2000, Dobryakova et al., 2017b, Lempert and Tricomi, 2015, Tricomi et al., 2006, Tricomi and Fiez, 2008, Tricomi and Fiez, 2012), both groups displayed greater activation in these regions in response to All Positive, relative to All Negative, Feedback.

Consistent with whole-brain analysis findings, *a priori* ROI analyses of the caudate and VS also revealed similar patterns of activity between MS and NT participants during All Positive and All Negative Feedback outcomes. After accounting for state CF, both groups showed increased responding of the VS to All Positive relative to All Negative Feedback, replicating past reports of VS sensitivity to feedback valence (e.g., Lempert & Tricomi, 2015). Both groups also displayed a more widespread reduction in caudate signaling during both All Positive and All Negative Feedback, which did not significantly differ by feedback valence. We did observe significant activation in the caudate in NT individuals after applying a more liberal cluster-defining threshold, however. One potential reason for this effect is the placement of the coordinates for the caudate seed, which reflect *a priori* (not peak) coordinates from Lempert & Tricomi (2015). Peak coordinates in that study resided within the ventral portion of the caudate (close to the VS). Since we were interested in both regions’ responses to feedback in the current study, we used the *a priori* coordinates to ensure adequate anatomical distinctiveness between the regions. The peak voxel from Lempert & Tricomi (2015) displays activation in the NT group at a cluster-defining threshold of α = 0.001 for this contrast in the present study. Taken together, these results suggest that MS participants recruit the striatum during feedback processing in a similar manner as NT participants.

### 4.4 Limitations and future directions

The present study contains a few limitations that should be considered. First, our work only provides evidence for intact feedback-based learning ability and its associated neural circuitry within the RRMS population. Other phenotypes of MS include more progressive disease courses, which display differences in cognitive impairment (Johnen et al., 2017), associated structural and functional neural circuitry (Cao et al., 2021, De la Cruz et al., 2021), and susceptibility to CF (Marchesi et al., 2020). An avenue for future work is to investigate feedback-based learning in progressive forms of MS. The present study also indicates that feedback-based learning ability and cognitive performance remain intact within the domain of verbal associative memory (or at least how it is assessed with this task). A potentially rich area of future work is the investigation of whether intact feedback-based learning replicates in other cognitive domains affected by MS. Finally, neuropsychological testing was conducted after the task, which may have influenced performance on these clinical assessments. However, there were no group differences on any measure, making it unlikely that task fatigue impacted testing.

## 5 Conclusions

In sum, we demonstrate that neither elevated trait CF nor elevated state CF disrupts the ability to learn from feedback related to associative memory performance in people with MS. MS and NT participants displayed comparable task performance and similar recruitment of the VS, caudate, and vmPFC during the processing of feedback – suggesting that feedback-based learning might be a cognitive capacity that is spared by CF in MS. Where these populations appear to differ, however, are the functional connections among these regions. During positive feedback outcomes, strengthening of VS-AG-STG connectivity in MS participants displayed potential associations with learning, providing a circuit of interest for future investigations into associative memory in MS. MS participants also displayed potential reductions in caudate-dACC connectivity associated with their elevated state CF, providing another circuit to be further examined in future studies on CF’s impact on learning. These findings may also have translational implications for performance during feedback-based treatments used in cognitive rehabilitation (Dardiotis et al., 2018, Hart et al., 2019, Whyte et al., 2019), and it is our hope that this line of research will optimize these treatments to maximize rehabilitation success.

## Funding

This work was supported by The National Multiple Sclerosis Society [Grant No. RG-1501–02630; PI: Dobryakova] and the National Science Foundation [Grant No. BCS 1756065; PI: Tricomi].

## CRediT authorship contribution statement

**Christopher J. Cagna:** Methodology, Validation, Formal analysis, Data curation, Writing – original draft, Writing – review & editing, Visualization. **Ahmet O. Ceceli:** Methodology, Writing – review & editing. **Joshua Sandry:** Methodology, Writing – review & editing. **Jamil P. Bhanji:** Methodology, Writing – review & editing. **Elizabeth Tricomi:** Methodology, Writing – review & editing, Supervision, Funding acquisition. **Ekaterina Dobryakova:** Conceptualization, Methodology, Resources, Writing – review & editing, Supervision, Project administration, Funding acquisition.

## Declaration of Competing Interest

The authors declare that they have no known competing financial interests or personal relationships that could have appeared to influence the work reported in this paper.

## Data Availability

Data will be made available on request.
