# Supplementary material for: Altered functional connectivity during performance feedback processing in multiple sclerosis
Source: Neuroimage Clin. 2022 Dec 7;37:103287. doi: 10.1016/j.nicl.2022.103287 (PMC9755233; doi:10.1016/j.nicl.2022.103287)
Supplement: Supplementary data 1 [file mmc1.docx]

**Supplementary Material**

**Materials and methods**

*Participants*

Three participants (1 MS, 2 NT) were excluded from data analysis due to technical error during fMRI data acquisition. Technical error during behavioral task data collection also occurred for two NT participants, excluding them from relevant analyses. Therefore, the final sample consisted of 57 participants (29 MS and 28 NT), while behavioral analyses consisted of 55 participants (29 MS and 26 NT). Neuropsychological data were unavailable for one NT participant, limiting relevant analyses to 54 participants (29 MS and 25 NT).

*Neuropsychological testing*

The battery consisted of the Brief Visuospatial Memory Test – Revised (BVMT-R; Benedict et al., 1996); the Wechsler Abbreviated Scale of Intelligence, Second Edition (WASI-II) Vocabulary Test (Wechsler, 2011); the WASI-II Matrix Reasoning Test (Wechsler, 2011); the Symbol Digit Modalities Test (SDMT) – Oral Version (Smith, 1982); the N-Back Test (Kirchner, 1958); the Wechsler Memory Scale, Fourth Edition (WMS-IV) Verbal Paired Associates Test (Wechsler, 2009); and the Ambulation Index (Hauser et al., 1983).

*Post-task questionnaire battery*

Other questionnaires in the battery included the Modified Fatigue Impact Scale (Fisk et al., 1994)], Behavioral Inhibition/Behavioral Activation Scale (Carver & White, 1994), Intrinsic Motivation Inventory (Ryan, 1982), Situational Motivation Scale (Guay et al., 2000), and Chicago Multiscale Depression Inventory (Nyenhuis & Luchetta, 1998).

*fMRI data analysis*

All images were skull-stripped and then motion-corrected. We used FSL’s Motion Outliers algorithm, which estimates variance from large deviations in head movement and generates a confound matrix of outliers to be included within the general linear model (GLM) as nuisance regressors. Blood-oxygen-level-dependent (BOLD) data were spatially smoothed using a Gaussian kernel of 5 mm full width at half maximum (FWHM), and a high-pass filter cutoff of 100 seconds was applied to minimize effects of residual signal. Functional data were then registered to Montreal Neurological Institute (MNI) standard space through a two-step process. Each participant’s functional data was co-registered to their anatomical data using FSL’s Boundary-Based Registration (BBR) algorithm, which accounted for large deviations in typical gray matter-white matter boundaries (Greve & Fischl, 2009) that were more likely to occur in the brains of MS participants as a result of disease-related morphological changes. The resulting image was then registered to standard MNI space using non-linear registration with a warp resolution of 10 mm.

*Table S1.* Linear mixed model of state fatigue (VAS-F score) responses.

| Fixed Effects | Estimate (*β)*  [95% CI] | *SE*_β_ | *t* | Sig. (*p*) |
| --- | --- | --- | --- | --- |
| **Intercept** | **2.66**  **[1.83, 3.49]** | **0.42** | **6.29** | **< .001** |
| TOT | 0.02  [-0.01, 0.06] | 0.02 | 1.31 | .19 |
| MF vs. NF | -0.10  [-0.31, 0.10] | 0.11 | -0.99 | .32 |
| NMF vs. NF | -0.08  [-0.28, 0.12] | 0.10 | -0.75 | .45 |
| **Group** | **1.86**  **[0.72, 3.00]** | **0.58** | **3.20** | **.002** |
| Random Effect (Participant) | | | | |
|  | Variance | SD |  |  |
| Intercept | 4.68 | 2.16 |  |  |
|  |  |  |  |  |
| TOT | 0.01 | 0.12 |  |  |
|  |  |  |  |  |
| Residual | 1.09 | 1.04 |  |  |
| Model Comparisons | | | | |
| Model | LL | *X^2^* | Sig. (*p*) |  |
| **Group (Null)** | **-1375.30** |  |  |  |
| **Group (Full)** | **-1370.40** | **9.71** | **.002** |  |
| TOT (Null) | -1371.30 |  |  |  |
| TOT (Full) | -1370.40 | 1.73 | .19 |  |
|  |  |  |  |  |
| MF vs. NF (Null) | -1370.90 |  |  |  |
| MF vs. NF (Full) | -1370.40 | 0.99 | .32 |  |
|  |  |  |  |  |
| NMF vs. NF (Null) | -1370.70 |  |  |  |
| NMF vs. NF (Full) | -1370.40 | 0.57 | .45 |  |

Note: TOT = Time-on-Task; MF = Monetary Feedback; NMF = Non-Monetary Feedback; NF = No Feedback; 95% CI = 95% confidence interval; SE = standard error; SD = standard deviation; LL = log-likelihood; Sig. = Significance. For each predictor, likelihood-ratio tests compared models containing that predictor (i.e., Full) with an otherwise identical model that did not contain the predictor (i.e., Null) to assess goodness of fit with the state fatigue data. Significant chi-square results indicate better fit of a Full, compared to a Null, model for that predictor.

*Table S2.* Linear mixed model of learning between Phases 2 and 3 (i.e., difference in percentage of correctly recalled responses between Phases 2 and 3).

| Fixed Effects | Estimate (b*)*  [95% CI] | *SE_b_* | *t* | Sig. (*p*) |
| --- | --- | --- | --- | --- |
| Intercept | -0.04  [-0.08, 0.01] | 0.02 | -1.48 | .14 |
| MF State Fatigue | -0.01  [-0.04, 0.02] | 0.02 | -0.64 | .52 |
| NMF State Fatigue | 0.01  [-0.02, 0.04] | 0.02 | 0.59 | .56 |
| NF State Fatigue | 0.004  [-0.02, 0.03] | 0.01 | 0.29 | .77 |
| Group | 0.03  [-0.01, 0.08] | 0.02 | 1.38 | .17 |
| **MF vs. NF** | **0.07**  **[0.04, 0.09]** | **0.01** | **5.29** | **< .001** |
|  |  |  |  |  |
| **NMF vs. NF** | **0.07**  **[0.04, 0.09]** | **0.01** | **5.51** | **< .001** |
|  |  |  |  |  |
| **Group x (MF vs. NF)** | **-0.05**  **[-0.08, -0.01]** | **0.02** | **-2.71** | **.007** |
|  |  |  |  |  |
| Group x (NMF vs. NF) | -0.03  [-0.07, 0.004] | 0.02 | -1.70 | .09 |
| Random Effect (Participant) | | | | |
|  | Variance | SD |  |  |
| Intercept | 0.004 | 0.06 |  |  |
| Residual | 0.005 | 0.07 |  |  |
| Model Comparisons | | | | |
| Model | LL | *X^2^* | Sig. (*p*) |  |
| MF State Fatigue (Null)  MF State Fatigue (Full) | 376.01  376.24 | 0.45 | .50 |  |
|  |  |  |  |  |
| NMF State Fatigue (Null)  NMF State Fatigue (Full) | 376.05  376.24 | 0.38 | .54 |  |
|  |  |  |  |  |
| NF State Fatigue (Null)  NF State Fatigue (Full) | 376.19  376.24 | 0.09 | .76 |  |
|  |  |  |  |  |
| Group (Null) | 372.47 |  |  |  |
| Group (Full) | 376.24 | 7.55 | .06 |  |
| **MF vs. NF (Null)** | **362.00** |  |  |  |
| **MF vs. NF (Full)** | **376.24** | **28.48** | **< .001** |  |
|  |  |  |  |  |
| **NMF vs. NF (Null)** | **357.94** |  |  |  |
| **NMF vs. NF (Full)** | **376.24** | **36.61** | **< .001** |  |
|  |  |  |  |  |
| **Group x (MF vs. NF) (Null)** | **372.58** |  |  |  |
| **Group x (MF vs. NF) (Full)** | **376.24** | **7.33** | **.007** |  |
|  |  |  |  |  |
| Group x (NMF vs. NF) (Null) | 374.79 |  |  |  |
| Group x (NMF vs. NF) (Full) | 376.24 | 2.90 | .09 |  |

Note: MF = Monetary Feedback; NMF = Non-Monetary Feedback; NF = No Feedback; 95% CI = 95% confidence interval; SE = standard error; SD = standard deviation; LL = log-likelihood; Sig. = Significance. For each predictor, likelihood-ratio tests compared models containing that predictor (i.e., Full) with an otherwise identical model that did not contain the predictor (i.e., Null) to assess goodness of fit with the delta performance data. Significant chi-square results indicate better fit of a Full, compared to a Null, model for that predictor.

*Table S3.* Linear mixed model of Phase 3 performance, accounting for valence of feedback received during Phase 2.

| Fixed Effects | Estimate (b*)*  [95% CI] | *SE_b_* | *t* | Sig. (*p*) |
| --- | --- | --- | --- | --- |
| **Intercept** | **0.55**  **[0.45, 0.65]** | **0.05** | **10.84** | **< .001** |
| MF State Fatigue | -0.01  [-0.08, 0.05] | 0.03 | -0.39 | .70 |
| NMF State Fatigue | 0.01  [-0.06, 0.07] | 0.03 | 0.24 | .81 |
| **Phase 2**  **Feedback Valence** | **0.21**  **[0.15, 0.26]** | **0.03** | **6.89** | **< .001** |
| Group | 0.03  [-0.06, 0.12] | 0.05 | 0.64 | .53 |
|  |  |  |  |  |
| Group x Phase 2  Feedback Valence | 0.01  [-0.07, 0.10] | 0.04 | 0.31 | .76 |
| Random Effect (Participant) | | | | |
|  | Variance | SD |  |  |
| Intercept | 0.01 | 0.11 |  |  |
| Residual | 0.01 | 0.11 |  |  |
| Model Comparisons | | | | |
| Model | LL | *X^2^* | Sig. (*p*) |  |
| MF State Fatigue (Null)  MF State Fatigue (Full) | 55.86  55.94 | 0.16 | .69 |  |
|  |  |  |  |  |
| NMF State Fatigue (Null)  NMF State Fatigue (Full) | 55.91  55.94 | 0.06 | .80 |  |
|  |  |  |  |  |
| **Phase 2 Feedback Valence (Null)**  **Phase 2 Feedback Valence (Full)** | **27.58**  **55.94** | **56.72** | **< .001** |  |
|  |  |  |  |  |
| Group (Null) | 55.47 |  |  |  |
| Group (Full) | 55.94 | 0.93 | .63 |  |
|  |  |  |  |  |
| Group x Phase 2 Feedback Valence (Null) | 55.89 |  |  |  |
| Group x Phase 2 Feedback Valence (Full) | 55.94 | 0.10 | .75 |  |
|  |  |  |  |  |

Note: MF = Monetary Feedback; NMF = Non-Monetary Feedback; 95% CI = 95% confidence interval; SE = standard error; SD = standard deviation; LL = log-likelihood; Sig. = Significance. For each predictor, likelihood-ratio tests compared models containing that predictor (i.e., Full) with an otherwise identical model that did not contain the predictor (i.e., Null) to assess goodness of fit with the Phase 3 performance data. Significant chi-square results indicate better fit of a Full, compared to a Null, model for that predictor.

*Table S4. Whole-brain Analysis Activation Clusters and Local Maxima for Contrasts Between Valenced Feedback and No Feedback*

|  |  |  |  |  | **MNI Coordinates (mm)** | | |
| --- | --- | --- | --- | --- | --- | --- | --- |
| **Cluster Number** |  | **Brain Region (Hemisphere)** | **Cluster Size (Voxels)** | **Z-statistic** | **x** | **y** | **z** |

**Whole-Brain Analysis**

***All Positive Feedback > No Feedback, MS***

| 1 |  | Occipital cortex (R) | 248 | 4.34 | 34 | -90 | -4 |
| --- | --- | --- | --- | --- | --- | --- | --- |

***All Positive Feedback > No Feedback, NT***

| 1 |  | Occipital cortex (R) | 4424 | 5.15 | 30 | -88 | -10 |
| --- | --- | --- | --- | --- | --- | --- | --- |
|  |  | Occipital cortex (L) |  | 4.98 | -28 | -92 | 2 |
| 2 |  | Thalamus (R) | 1485 | 4.96 | 4 | -12 | 12 |
|  |  | Caudate (L) |  | 4.49 | -8 | 12 | 0 |
| 3 |  | Dorsal anterior cingulate cortex (R) | 239 | 4.82 | 2 | 44 | 2 |
|  |  | Paracingulate gyrus (L) |  | 3.54 | -2 | 54 | 12 |
|  |  | Paracingulate gyrus (R) |  | 3.52 | 2 | 44 | 16 |
|  |  | Dorsal anterior cingulate cortex (L) |  | 3.23 | -8 | 38 | 14 |
| 4 |  | Anterior prefrontal cortex (L) | 140 | 3.64 | -4 | 66 | 24 |
| 5 |  | Inferior frontal gyrus, pars opercularis (R) | 104 | 4.20 | 48 | 10 | 22 |
| 6 |  | Cerebellum (R) | 103 | 4.48 | 52 | -64 | -38 |

***All Negative Feedback > No Feedback, NT***

| 1 |  | Insula (R) | 486 | 4.98 | 40 | 18 | 0 |
| --- | --- | --- | --- | --- | --- | --- | --- |
|  |  | Inferior frontal gyrus, pars triangularis (R) |  | 3.84 | 36 | 30 | 4 |
| 2 |  | Insula (L) | 309 | 5.24 | -36 | 16 | -8 |
|  |  | Frontal operculum cortex (L) |  | 3.81 | -42 | 16 | 2 |
| 3 |  | Superior frontal gyrus (R) | 295 | 4.37 | 10 | 18 | 64 |
|  |  | Superior frontal gyrus (L) |  | 3.97 | -4 | 12 | 54 |
|  |  | Paracingulate gyrus (L) |  | 3.81 | -6 | 18 | 46 |
|  |  | Supplementary motor area (L) |  | 3.79 | -4 | 8 | 60 |
| 4 |  | Paracingulate gyrus (R) | 271 | 4.62 | 10 | 30 | 34 |
|  |  | Dorsal anterior cingulate cortex (L) |  | 4.09 | -6 | 30 | 32 |
|  |  | Dorsolateral prefrontal cortex (R) |  | 3.62 | 8 | 38 | 26 |
| 5 |  | Precentral gyrus (R) | 155 | 4.69 | 54 | -4 | 42 |

***All Negative Feedback < No Feedback, NT***

| 1 |  | Ventromedial prefrontal cortex (L) | 600 | 4.30 | -14 | 46 | -6 |
| --- | --- | --- | --- | --- | --- | --- | --- |
|  |  | Subcallosal cortex (L) |  | 4.26 | -6 | 26 | -10 |
|  |  | Orbitofrontal cortex (L) |  | 4.06 | -4 | 38 | -16 |
| 2 |  | Precuneus (R) | 85 | 4.10 | 4 | -54 | 22 |

Peak intensities within each cluster with z values exceeding the cluster-defining threshold of 3.1 (p < .001, corrected to p < .05) for each event/contrast of events. Each unique cluster identified within the event/contrast is numerically listed in the “Cluster Number” column. When multiple peaks were identified within the same region, the reported coordinates correspond to peak activation with the highest z value. The Harvard-Oxford and Montreal Neurological Institute (MNI) Structural Atlases were used for the identification of anatomical brain region labels. MS = multiple sclerosis, NT = neurotypical, L = left hemisphere, R = right hemisphere.


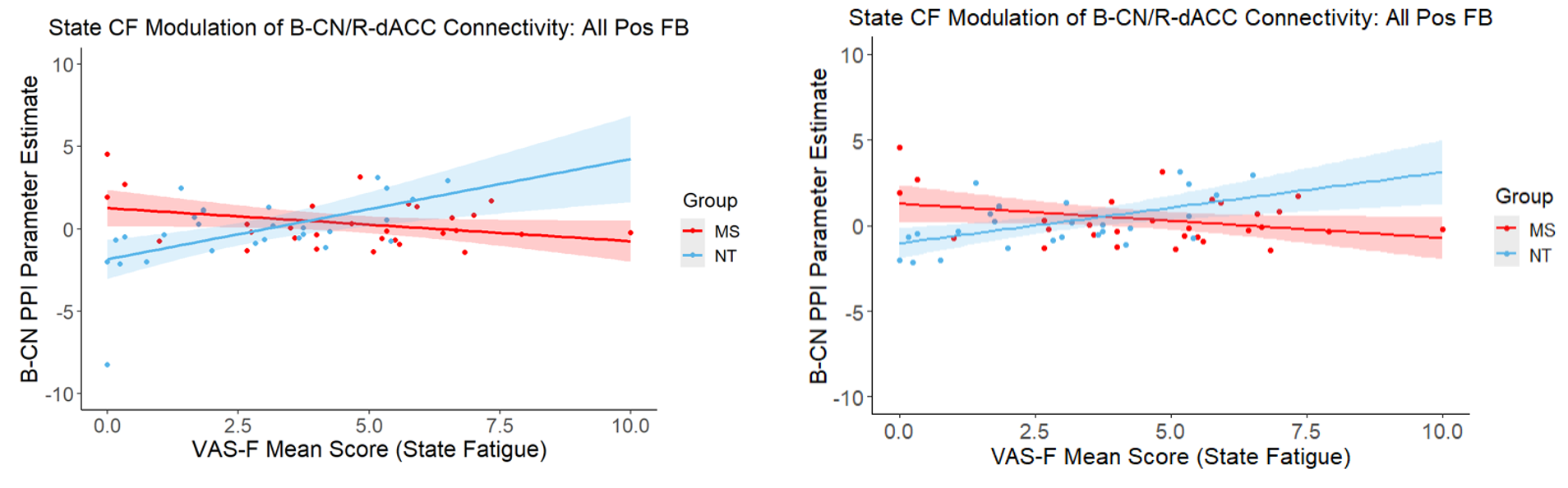


*Figure S1.* Group interaction between caudate-dACC functional connectivity and state CF with a NT outlier included (left panel) and excluded (right panel). Group level means and 95% confidence intervals are displayed in the plots. MS = multiple sclerosis; NT = neurotypical; B-CN = bilateral caudate nucleus; R-DACC = right dorsal anterior cingulate cortex.


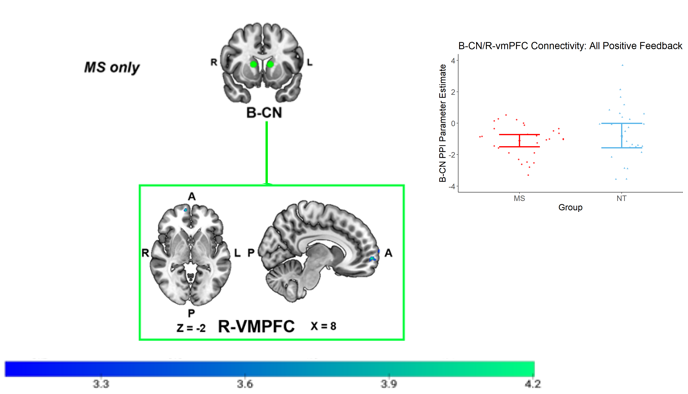


*Figure S2.* Within MS participants, the caudate displayed reduced connectivity with the right vmPFC during All Positive Feedback outcomes. Group level means and 95% confidence intervals are displayed in the plot. A cluster-defining threshold of *z* = 3.1 (α = .001) and a cluster-extent correction of *α* = .05 (uncorrected for multiple comparisons between models) were applied in this analysis. Cooler colors in the contrast map correspond to reduced functional connectivity. MS = multiple sclerosis; B-CN = bilateral caudate nucleus; R-vmPFC = right ventromedial prefrontal cortex.

References

Benedict, R. H. B., Schretlen, D., Groninger, L., Dobraski, M., & Shpritz, B. (1996). Revision of the Brief Visuospatial Memory Test: Studies of normal performance, reliability, and validity. *Psychological Assessment, 8*(2), 145-153. <https://psycnet.apa.org/doi/10.1037/1040-3590.8.2.145>

Carver, C. S., & Whitgrevee, T. L. (1994). Behavioral inhibition, behavioral activation, and affective responses to impending reward and punishment: The BIS/BAS scales. *Journal of Personality and Social Psychology, 67*, 319-333. <https://psycnet.apa.org/doi/10.1037/0022-3514.67.2.319>

Fisk, J.D., Ritvo, P. G., Ross, L., Haase, D. A., Marrie, T. J., & Schlech, W. F. (1994). Measuring the functional impact of fatigue: Initial validation of the fatigue impact scale. *Clinical Infectious Diseases, 18*, S79-S83. <https://doi.org/10.1093/clinids/18.Supplement_1.S79>

Greve, D. N., & Fischl, B. (2009). Accurate and robust brain image alignment using boundary-based registration. *NeuroImage*, *48*(1), 63-72. <https://doi.org/10.1016/j.neuroimage.2009.06.060>

Guay, F., Vallerand, R. J., & Blanchard, C. (2000). On the assessment of situational intrinsic and extrinsic motivation: The Situational Motivation Scale (SIMS). *Motivation and Emotion, 24*(3), 175-213. <https://doi.org/10.1023/A:1005614228250>

Hauser, S. L., Dawson, D. M., Lehrich, J. R., Beal, M. F., Kevy, S. V., Propper, R. D., Mills, J. A., & Weiner, H. L. (1983). Intensive immunosuppression in progressive multiple sclerosis – A randomized, three-arm study of high-dose intravenous cyclophosphamide, plasma exchange, and ACTH. *The New England Journal of Medicine, 308*(4), 173-180. <https://doi.org/10.1056/NEJM198301273080401>

Kirchner, W. K. (1958). Age differences in short-term retention of rapidly changing information. *Journal of Experimental Psychology*, 55(4), 352-358. <https://psycnet.apa.org/doi/10.1037/h0043688>

Nyenhuis, D. L., & Luchetta, T. (1998). The development, standardization, and initial validation of the Chicago Multiscale Depression Inventory. *Journal of Personality Assessment*, *70*, 386-401. <https://doi.org/10.1207/s15327752jpa7002_14>

Ryan, R. M. (1982). Control and information in the intrapersonal sphere: An extension of cognitive evaluation theory. *Journal of Personality and Social Psychology, 43*(3), 450-461. <https://psycnet.apa.org/doi/10.1037/0022-3514.43.3.450>

Smith, A. (1982). *Symbol Digit Modalities Test: Manual.* Los Angeles, CA: Western Psychological Services.

Wechsler, D. (2009). *Wechsler Memory Scale, Fourth Edition (WMS-IV)*. San Antonio, TX: Pearson.

Wechsler, D. (2011). *Wechsler Abbreviated Scale of Intelligence, Second Edition (WASI-II)*. San Antonio, TX: Pearson.
